# The Prognostic Value of Distinct Histological Growth Patterns of Colorectal Peritoneal Metastases: A Pilot Study

**DOI:** 10.1245/s10434-023-13118-x

**Published:** 2023-02-08

**Authors:** Antoine El Asmar, Pieter Demetter, Fahd Fares, Francesco Sclafani, Alain Hendlisz, Vincent Donckier, Peter Vermeulen, Gabriel Liberale

**Affiliations:** 1 Department of Surgical Oncology, Institut Jules Bordet, Université Libre de Bruxelles, Brussels, Belgium; 2 Department of Pathology, Institut Jules Bordet, Université Libre de Bruxelles, Brussels, Belgium; 3 Department of Surgery, Université Libre de Bruxelles, Brussels, Belgium; 4 Department of Medical Oncology, Institut Jules Bordet, Université Libre de Bruxelles, Brussels, Belgium; 5 Translational Cancer Research Unit, Department of Oncological Research, Oncology Center GZA, GZA Hospitals St. Augustinus, and University of Antwerp, Antwerp, Belgium

## Abstract

**Background:**

Different histological growth patterns (HGP) describing the tumor-to-liver interface have been described in colorectal liver metastases and have been associated with a strong prognostic value. However, HGP of peritoneal metastases (PM) of colorectal cancer (CRC) have not yet been described. Our objective was to determine whether distinct HGP can be identified in PMCRC and to evaluate their potential prognostic value in these patients.

**Methods:**

This retrospective study included 38 patients who underwent curative-intent surgery for PMCRC between July 2012 and March 2019, with PCI≤6, and who had not received preoperative chemotherapy. In each patient, the tumor-to-peritoneum interface was evaluated in the excised peritoneal nodules. The association between HGP and postoperative survival was analyzed by using the Kaplan–Meier method.

**Results:**

Two distinct HGP were identified: a *pushing*-type (P-HGP), characterized by a fibrous rim separating the PM and peritoneum, and an *infiltrating*-type (I-HGP), characterized by focal penetration of tumor cells into the surrounding peritoneal lining without a fibrous rim. Fifteen patients had dominant P-HGP, and 23 patients had dominant I-HGP. Patients with dominant P-HGP (>50% tumor-peritoneum interface) had a significantly better DFS (30 months) than those with P-HGP <50% (9 months; *p* = 0.029). Patients with a P-HGP dominance >60% had better OS (131 months) than those with P-HGP <60% (41 months; *p* = 0.044).

**Conclusions:**

This is the first description of two distinct, reproducible HGP in PMCRC. The dominant P-HGP is associated with a favorable prognosis in patients with PMCRC, compared with I-HGP, suggesting that this parameter could ultimately represent a new prognostic biomarker.

Metastatic colorectal cancer (CRC) is one of the leading causes of cancer-related deaths worldwide.^1^ Approximately 15% and 7% of these patients present with synchronous hepatic metastases (HM)^1,2^ and peritoneal metastases (PM), respectively.^3^ Furthermore, an additional 16–20% will develop metachronous HM within 3 years after the diagnosis, and up to 19% will develop PM, even after curative-intent surgery.^1,3–5^

Whether dealing with liver or peritoneal metastasis, characteristics of the primary tumors and of the metastatic lesions are used to predict the outcome of patients with CRC undergoing surgery.^6^ Given the high metastatic incidence of CRC, affecting approximately 65% of patients throughout the course of the disease,^1^ many studies are being conducted to optimize the treatment approach for these patients.^7, 8^ While HM of colorectal origin (HMCRC) are usually treated by resection of the liver lesions whenever feasible,^4^ limited PM often are managed with resection of all the peritoneal metastatic deposits, an intervention known as cytoreductive surgery (CRS), with or without hyperthermic intraperitoneal chemotherapy (HIPEC).^9^ Various treatment plans for HM and PM are still being studied in order to devise the optimal timing and type of surgical intervention in these patients or to combine the adequate chemotherapy agents, according to patient and tumor characteristics.^9–13^

In the absence of accurate predictive and prognostic markers in patients who are candidates for surgery for HMCRC or peritoneal metastases of CRC origin (PMCRC), the outcome in these cases remains extremely heterogeneous and poorly predictable. Strikingly, the majority of these patients experienced recurrence postoperatively, including a significant proportion of patients with rapid and aggressive relapse. Accordingly, the identification of new markers of metastatic behavior in patients with HMCRC or PMCRC would represent significant progress to better individualize therapeutic management.

Recently, the histopathological growth pattern (HGP) of colorectal liver metastases has been reported to be a major prognostic factor in patients undergoing HM resection.^14–22^ Three distinct HGP have been described in consensus scoring guidelines: the desmoplastic HGP, where the metastatic lesion is surrounded by a collagen rim with lymphocytic infiltration, separating it from the liver parenchyma; the *pushing* HGP, where the metastatic tumor cells push the liver parenchyma, exerting pressure on nearby hepatocytes; and the replacement HGP, where tumor cells replace the hepatocytes, while maintaining the trabecular architecture of the liver parenchyma and performing vessel co-option and not angiogenesis.^23,24^ It has now been reproducibly shown that patients with a desmoplastic HGP have a better prognosis in terms of overall survival, progression-free survival, and recurrence-free survival, compared with the nondesmoplastic group.^16,18,19,21,25,26^ Galjart et al. reported a favorable prognosis of desmoplastic growth patterns in chemo-naive patients, as well as in those who had received neoadjuvant therapy.^25^

Although HGP has been extensively studied in HMCRC, it has never been reported in PMCRC. The existence of reproducible HGP in PMCRC is yet to be demonstrated, as is its implication in terms of prognosis in patients who underwent CRS with or without HIPEC.

In this study, we aimed to identify the different HGP in patients with PMCRC who underwent CRS with or without HIPEC and to investigate the impact of this parameter on disease-free survival (DFS) and overall survival (OS).

## Materials and Methods

### Design

We retrospectively reviewed all patients who underwent surgery with curative intent (R0/R1 resection) CRS (± HIPEC) for PMCRC at our institution between July 2012 and March 2019. The study was approved by the Ethics Committee (CE3222).

### Patient Selection

Only patients who had an intraoperative peritoneal cancer index (PCI) ≤6 and who did not receive neoadjuvant chemotherapy were included. Most of these patients were evaluated by morphologic imaging, including abdominopelvic computed tomography and/or nuclear magnetic resonance, and by FDG-PET-scan to rule out distant metastases.

### Pathology

All pathology reports from the operative specimens of the eligible patients were reviewed. Two of the largest, completely excised peritoneal nodules were chosen for each patient. The corresponding pathology slides were assessed for HGP by two pathologists (PD and PV). Slides showing the largest circumferential margins between the tumor and the peritoneal/subperitoneal tissues were analyzed. Peritoneal nodules confined within a resected organ (e.g., spleen, liver, and ovary) were excluded. The analysis was limited to nodules with direct contact with the peritoneal surface. The tumor periphery was assessed for HGP on hematoxylin and eosin (H&E)-stained slides. Cases where the PM consisted only of fibrotic and/or necrotic tissue or when the tumor-peritoneum interface could not be analyzed were considered nonassessable (NA) and were excluded from further analysis. HGP evaluation was performed by using a Leica bright-field microscope at a low magnification (10x objective). For each slide, the relative presence (expressed as a percentage) of the different HGP at the tumor-peritoneal interface was estimated. Then, the relative proportion of each HGP from the total interface length was calculated for each nodule. The mean HGP scores were calculated for the two nodules from each patient.

### Statistical Analysis

Clinical data included patient demographics, primary tumor histological and molecular features, type of adjuvant chemotherapy (if any) before CRS, disease-free survival (DFS), and overall survival (OS). These were pseudonymized and merged into a study database using an Excel spreadsheet (Microsoft, Brussels, Belgium). Descriptive statistics were used to describe the study population and its characteristics (SPSS v27, IBM, New York, NY). The Student test or the Mann-Whitney *U* test were applied for continuous variables according to their distribution (normal or not). The Chi-square test was used for categorical variables. Overall survival was defined as the time interval between the date of CRS and the date of death from any cause or the last follow-up. Disease-free survival was defined as the time interval between the date of CRS and the date of first recurrence or death. Patients who were lost to follow-up were censored at the date they were known to be alive and disease free. The Kaplan–Meier (KM) method and log-rank test were used to calculate and compare the survival curves for DFS and OS in patients with different HGP. Univariate analysis was performed to evaluate prognostic factors.

Finally, to assess the analytical validity of our HGP scoring system, a discussion session was held with all co-authors. During this session, several slides were simultaneously evaluated by two pathologists with experience in liver HGP evaluation (PV and PD). Agreement was reached on the methodology of HGP scoring for PMCRC. Subsequently, two pathologists, blinded to the patient outcome (38 patients), were provided with a validation set comprising the slides of the involved patients (380 slides). The results of the validation study are tabulated with rows representing the different participants (PV and PD) and columns indicating the percentage of the interface occupied by each HGP. After obtaining the HGP scores (pushing versus infiltrating percentage for each nodule), an intraclass correlation coefficient (ICC) was used to measure the inter-rater agreement. ICC values <0.5 indicate poor reliability, values between 0.5 and 0.75 moderate reliability, values between 0.75 and 0.9 good reliability, and values >0.90 excellent reliability.

## Results

### Patients

We identified 94 patients who underwent complete CRS ± HIPEC for PM of CRC origin. Among these, 50 patients had PCI ≤6. Twelve additional patients were excluded: two had intraovarian metastasis only, eight had received neoadjuvant chemotherapy, and two had no available archived slides for analysis, resulting in 38 patients included in our study.

The patient characteristics are reported in Table 1. There were 18 males and 20 females, with median age of 62 (range, 40–76) years. Primary tumors were of colonic origin in 87% and rectal origin in 13% of patients. The median PCI was 4.

**Table 1:** Characteristics and prognostic impact of clinical, demographic, and histopathological factors in patients with PMCRC, presenting for cytoreductive surgery

Variable	N (%)	DFS		OS	
		Median DFS (range in month)	*p*	Median OS (range in month)	*p*
Age (year)	38 (100%)	14 (8.9–19.2)	0.945	54 (25.3–82.7)	0.161
>62	19 (50%)	14 (1.6–26.5)		85 (21.5–148.5)	
≤62	19 (50%)	9 (4.8–13.2)		51 (30.5–71.5)	
Gender	38 (100%)	14 (8.9–19.2)	0.858	54 (25.3–82.7)	0.829
Male	18 (47.4%)	14 (8.8–19.2)		51 (31.2–70.8)	
Female	20 (52.6%)	9 (6.8–11.2)		85 (35–135)	
MMR	28 (100%)	11 (6.1–16)	**0.012**	51 (34.4–67.6)	0.32
MSS	23 (82.1%)	9 (8–10.1)		51 (37.3–64.7)	
MSI	5 (17.9%)	–		–	
KRAS	28 (100%)	9 (5.3–12.7)	0.199	51 (35.1–66.9)	0.635
Wild	17 (60.7%)	9 (0.2–17.8)		51 (28.9–73.1)	
Mutated	11 (39.3%)	9 (3.6–14.4)		51 (34.4–67.6)	
Tumor grade differentiation	37 (100%)	14 (8.9–19.1)	**0.031**	54 (25.5–82.5)	0.873
Well	10 (27%)	11 (5.2–16.9)		51 (34.6–67.4)	
Moderately	19 (51.4%)	9 (5–13)		54 (25.5–82.5)	
Poorly	8 (21.6%)	42 (N/A)		–	
Lymph nodes status	38 (100%)	14 (8.9–19.1)	**0.012**	54 (25.3–82.7)	**0.014**
pN0	10 (26.3%)	131 (N/A)		131 (N/A)	
pN1	13 (34.2%)	9 (4.3–13.7)		51 (27.9–74.2)	
pN2	15 (39.5%)	9 (6.7–11.3)		35 (19.9–50.1)	
pT*	37 (100%)	14 (8.9–19.1)	0.93	54 (25.5–82.5)	0.882
3	18 (48.6%)	15 (7.3–22.8)		54 (28.2–79.8)	
4	19 (51.4%)	9 (1.5–16.5)		85 (N/A)	
Localization	38 (100%)	14 (8.9–19.1)	0.463	54 (25.3–82.7)	0.835
Colon	33 (86.8%)	15 (6.8–23.2)		54 (24–84)	
Rectum	5 (13.2%)	6 (N/A)		37 (4.3–69.7)	
Peritoneal metastases	38 (100%)	14 (8.9–19.1)	0.363	54 (25.3–82.7)	0.232
Metachronous	21 (55.3%)	15 (1.9–28.1)		131 (N/A)	
Synchronous	17 (44.7%)	8 (6.1–9.9)		51 (30.3–71.7)	
ASA*	37 (100%)	14 (8.9–19.1)	**0.038**	54 (25.5–82.5)	0.056
2	25 (67.6%)	19 (0–38.2)		85 (24.9–145.1)	
3	12 (32.4%)	9 (6.9–11.1)		38 (19–57)	
CEA	33 (100%)	15 (5.2–24.8)	0.832	54 (24.4–83.6)	0.729
≤5	18 (54.5%)	19 (1.5–36.5)		85 (0–171.9)	
>5	15 (45.45%)	11 (1.8–20.2)		51 (34.9–67.1)	
CA19.9	19 (100%)	14 (5.5–22.5)	0.544	41 (16.4–65.6)	0.839
≤37	14 (73.7%)	14 (6.7–21.3)		85 (18.8–63.2)	
>37	5 (26.3%)	7 (4.9–9.1)		41 (N/A)	
PCI	38 (100%)	14 (8.9–19.1)	0.797	54 (25.3–82.7)	0.839
≤4	26 (68.4%)	15 (5.3–24.7)		51 (36.6–65.4)	
>4	12(31.6%)	9 (4.7–13.3)		85 (N/A)	
Liver metastasis	38 (100%)	14 (8.9–19.1)	**0.001**	54 (25.3–82.7)	**0.025**
No	21 (55.3%)	22 (0–67)		131 (N/A)	
Yes	17 (44.7%)	8 (5.6–10.4)		37 (26.3–47.7)	
Size of nodule 1 (mm)	28 (100%)	9 (6–12)	0.901	51 (34.1–67.9)	0.982
≤18	15 (53.6%)	9 (5.5–12.5)		85 (N/A)	
>18	13 (46.4%)	9 (2.8–15.2)		51 (35–67)	
Size of nodule 2	21 (100%)	9 (4.6–13.4)	0.519	51 (16.9–85.1)	0.296
≤ 20 mm	12 (57.1%)	8 (0–21.6)		85 (20.8–149.2)	
> 20 mm	9 (42.9%)	9 (6.3–11.7)		38 (25.4–50.6)	
HGP^‡^	38 (100%)	14 (8.9–19.1)	**0.029**	54 (25.3–82.7)	**0.044**
P-HGP					
<50%	23 (60.5%)	9 (6.7–11.3)		N/A	
<60%	26 (68.4%)	N/A		41 (27–55)	
P-HGP					
>50%	15 (39.5%)	30 (0–81.1)		N/A	
≥60%	12 (31.6%)	N/A		131 (N/A)	

Clinically Significant *p*-values (< 0.05) given in bold

*None of the patients in this series had pT1-2 primary tumor nor preoperative ASA 1 or 4 scores.

^**‡**^*HGP* Histological Growth Pattern, *P-HGP* Pushing-HGP. The significant dominant cutoff amongst the different HGPs was 50% for DFS and 60% for OS.

### Histological Growth Patterns of Peritoneal Metastases

In total, 66 peritoneal nodules were resected. Of these, 55 (83.3%) were chosen for analysis, and 11 nodules were discarded, because they lacked an adequate tumor-peritoneum interface. The median size was 18 mm for nodule 1 and 20 mm for nodule 2 in patients with a second nodule available for analysis. A total of 380 slides were reviewed, and 130 were considered contributive for HGP classification (harboring the largest circumferential margins between the metastatic lesion and peritoneum).

**Fig. 1:**
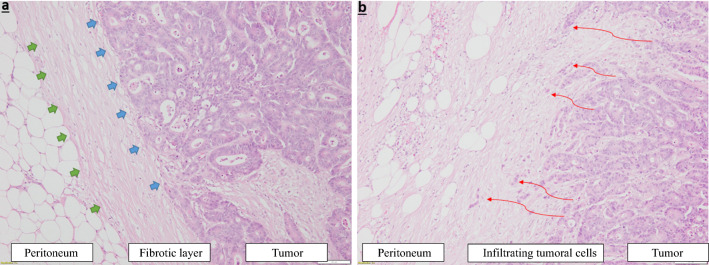
Two types of tumor-peritoneum interaction observed: **a** Fibrous rim surrounds the tumor separating it from healthy peritoneum with an absence of tumoral cells penetration into the peritoneal lining, entitled: the *Pushing Histological Growth Pattern*; **b** no fibrous rim surrounds the tumor, with focal penetration of tumoral cells into the peritoneal lining, entitled: the *Infiltrating Histological Growth Pattern*

Two main types of tumor-peritoneum interaction were observed (Fig. 1):

1. Absence of focal penetration of tumor cells into the surrounding peritoneal lining, where the healthy tissue is pushed back by a fibrous rim; this type is called *pushing*-HGP or P-HGP (Fig. 1a).
2. Focal penetration of tumor cells into the surrounding peritoneal lining, without a separating fibrous rim; this type is called *Infiltrating*-HGP or I-HGP (Fig. 1b).

A particular growth pattern was considered dominant whenever it demarcated ≥50% of the nodule-peritoneum interface for DFS and ≥60% for OS (the cutoff after which the dominant pattern became significantly correlated with OS).

**Fig. 2:**
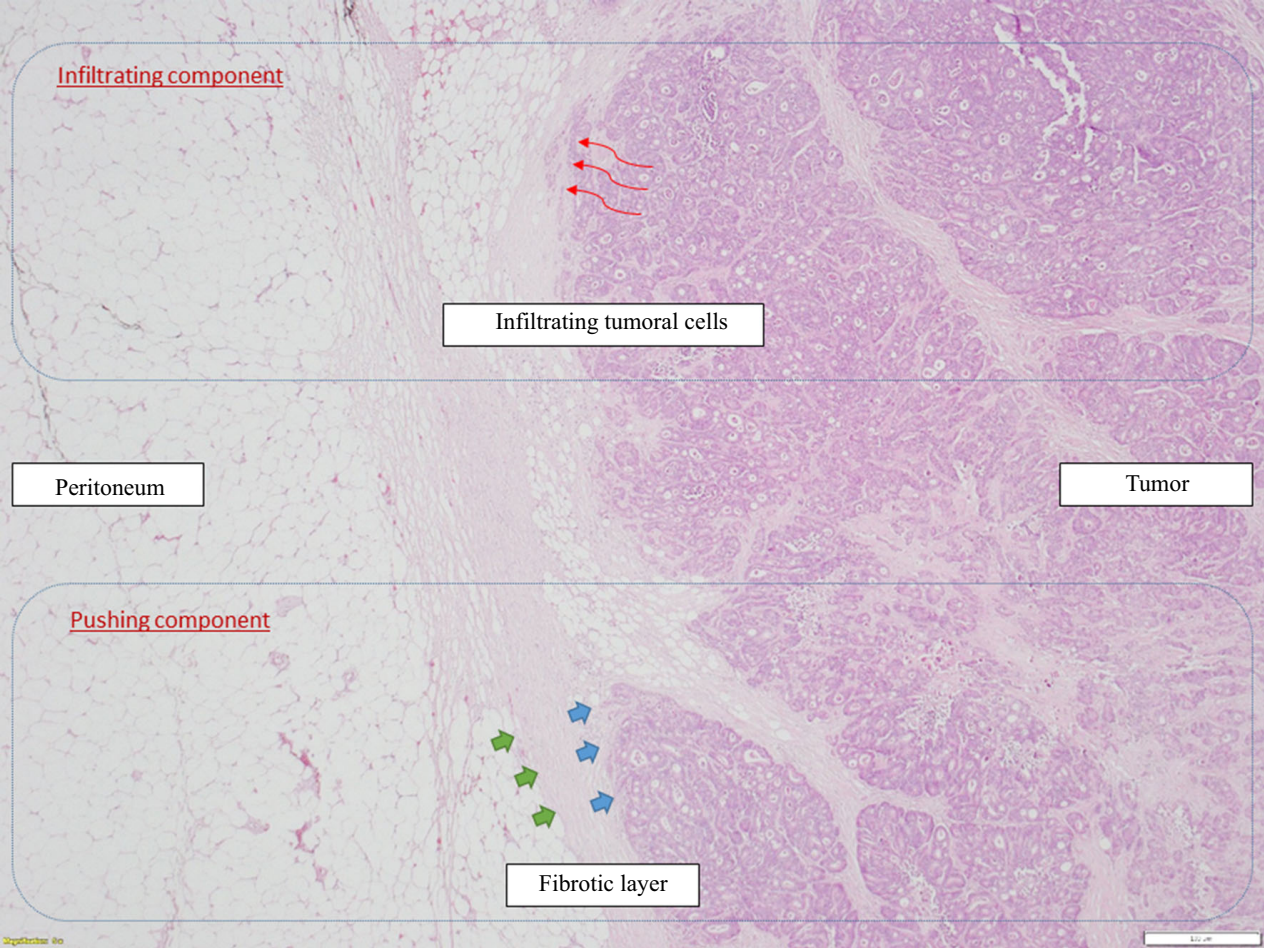
Colorectal peritoneal metastatic nodule, showing a mixed histological pattern: an *infiltrating* component (upper part), and a concomitant *pushing* component with a delineating fibrous ring (lower part)

**Fig. 3:**
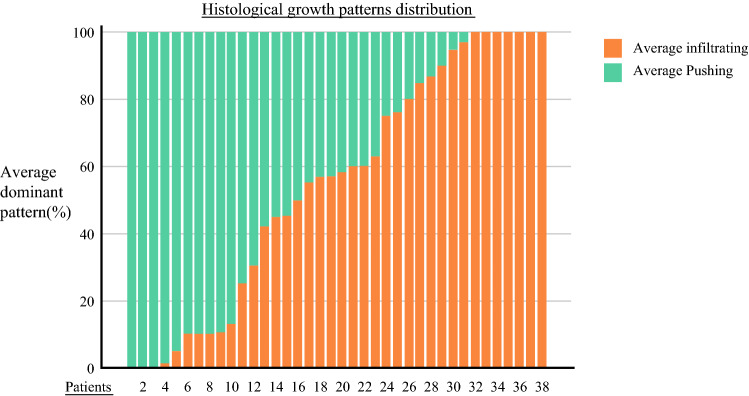
Histological growth patterns dominance and distribution in the whole population

A dominant P-HGP was found in the PM of 15 (39.5%) patients. A dominant I-HGP was found in the PM of 23 (60.5%) patients. The intrapatient distribution of HGP is shown in Fig. 2. Patterns of dominance in the entire population are shown in Fig. 3.

Overall, 18 patients had two assessable PMs. In only three cases (16.6%), the two PMs were found to have a different HGP: a *pushing* pattern was dominant in nodule 1 (70, 90%, and 95%), whereas the *infiltrating* pattern was dominant in nodule 2 (85, 80%, and 85%, respectively for the 3 patients).

### Interobserver Analysis

Once both pathologists read the images separately, the intraclass score was calculated as ICC = 0.978 (95% confidence interval [CI]: 0.883–0.996; *p* < 0.001). This score (>0.90) indicates excellent reliability. In other words, both pathologists were able to identify the same histological growth patterns, and their scores for each growth pattern on the nodule-peritoneal interface were extremely similar, if not similar, in some cases. This strengthens the fact that the identified HGPs had excellent reproducibility.

### Prognostic Factors for Disease-Free Survival and Overall Survival

The univariate analysis of prognostic factors for DFS and OS is reported in Table 1. Absence of locoregional lymph node (pN+) involvement, absence of synchronous hepatic metastases, and dominant P-HGP were favorable prognostic factors for DFS and OS. Moreover, the tumor grade (*p* = 0.031), ASA score (*p* = 0.038), and MSI status (*p* = 0.012) were predictors of DFS.

**Fig. 4:**
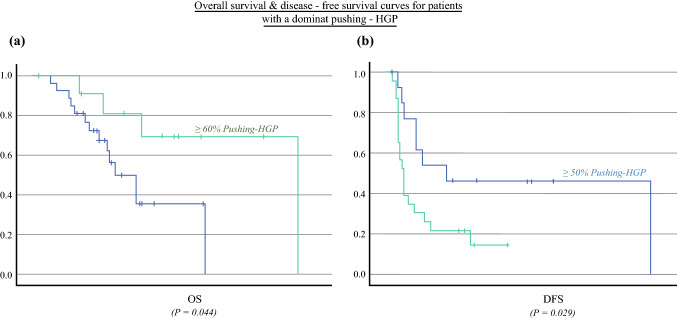
Overall and disease-free survival curves for patients with a dominant *Pushing-HGP* compared with patients with a nondominant pushing pattern

A >50% dominance of P-HGP was a predictor of better DFS compared with patients with a P-HGP <50%: a median of 30 versus 9 months, respectively, with *p* = 0.029. Furthermore, in patients in whom P-HGP dominance reached and exceeded 60% of the tumor-peritoneal interface, the median OS was better than in those with a P-HGP <60%: 131 versus 41 months, respectively (*p* = 0.044; Fig. 4). As shown previously, dominant P-HGP was significantly correlated with OS once it surpassed 60% versus 50% for DFS.

## Discussion

To our knowledge, this is the first study to analyze HGP in PMCRC patients. In this series, we observed that distinct HGP of PMCRC could be identified using the pathological characteristics of the tumor-peritoneum interface. In contrast to what has been described for HMCRC, only two HGP could be identified: P-HGP and I-HGP. Nevertheless, similar to the observations in HMCRC, these patterns of tumor growth are associated with prognosis in patients who underwent CRS ± HIPEC, as significantly better postoperative DFS and OS were observed in patients with dominant P-HGP than in patients with a dominant I-HGP.

In HMCCR, accumulating data suggest that different HGP could be associated with different types of metastatic biology and modes of progression. Besides its independent prognostic value in patients undergoing liver resection, it has been shown indeed that HGP of HMCRC also can predict the sensitivity to chemotherapy. Taken together, these data suggest that the histological morphology of the invasive front can accurately reflect host-cancer interactions associated with systemic metastatic behavior. At present, the determinant driver mechanisms of different HGP remain poorly understood. Factors, such as the molecular profile and histopathological characteristics of the primary tumor or the host immune reaction to metastatic deposits, are being evaluated in ongoing studies.^26–29^

In this study, we first observed that distinct HGP could be identified in PMCRC, similar to HMCRC. Two types of HGP were observed at the margin between the PMCRC and the peritoneal surface that we defined as the “*Pushing*” type, characterized by an absence of focal penetration of tumor cells into the surrounding tissue; and the “*Infiltrating*” type, characterized by focal penetration of tumor cells into the surrounding tissue. In contrast to HMCRC, none of these HGP exhibited prominent inflammatory cell infiltration at the tumor-peritoneum interface. Furthermore, in contrast to HMCRC, we found significant intralesion heterogeneity of HGP as peritoneal nodules in our study, which was characterized by the coexistence of different growth patterns.

These preliminary observations indicated that the HGP of PMCRC could represent a promising biomarker. We found that whenever the “Pushing” component exceeds 50%, this dominant HGP has a positive prognostic impact on DFS:30 months versus 9 months for patients with P-HGP <50%. Furthermore, whenever the “Pushing” component exceeded 60%, it conveys a favorable prognostic outcome on OS:131 months versus 41 months for patients with P-HGP <60%. This confirmed the contention that, as in the liver, the growth pattern could reflect distinct biological properties of the PMs. Additional studies to define these properties will be important to better understand the mechanisms of cancer progression in patients with PMCRC and improve their oncosurgical management.

Currently, HGP cannot be used as a biomarker to guide therapeutic decisions before the surgical removal of metastases in patients with HMCRC. Indeed, HGP scoring of liver metastases requires analysis of the entire tumor-to-liver interface, which is not possible in a biopsy. Therefore, several studies are ongoing to evaluate dedicated imaging techniques and radiomics algorithms for predicting the HGP of liver metastases.

Interestingly, this limitation can potentially be bypassed in patients with PM. Indeed, as diagnostic laparoscopy is generally performed before CRS to evaluate PCI and confirm the indication for surgery, peritoneal nodules could be sampled and evaluated for their HGP during such a procedure. Based on the first results in our study showing limited heterogeneity between nodules from the same patient, we might assume that the HGP analysis of biopsies of a limited number of PM could be representative of the entire tumor status, providing relevant information for patient-oriented therapeutic decision-making.

Our study had several limitations. In addition to the retrospective design of the study, these data should be considered preliminary and require further validation in larger cohorts. Importantly, our findings and their reproducibility must be verified in independent and multicenter studies, including other pathological experts in the field. At this stage, we only included patients with a limited disease burden (PCI ≤ 6) to allow a feasible evaluation of a representative part of the peritoneal disease in each case. In patients with PCI > 6, a higher number of PM is warranted; this population will be further studied (comprising a greater number of nodules) in our upcoming projects. Moreover, to avoid a potential bias in interpretation related to the influence of chemotherapy on HGP,^30^ we also excluded patients who received preoperative chemotherapy.

Therefore, our results must be confirmed in future studies, including those with higher PCI rates and those exposed to systemic chemotherapy before CRS. We cannot exclude the possibility that these variables may affect the overall distribution of HGP as well as their prognostic relevance. However, the strong inter-observer correlation we observed reflects a high level of reproducibility, permitting further studies on the subject to validate our findings and their prognostic impact in patients with PMCRC.

## Conclusions

PMCRC can express two distinct HGP that coexist in the same lesions in varying proportions. The dominant Pushing-HGP of PMCRC appears to be a major prognostic factor that reflects a more favorable postoperative oncological outcome in patients undergoing CRS ± HIPEC.

## Data Availability

Research data that support this publication are available upon Editor’s request.
